# Pharmacometabolomic profiles in type 2 diabetic subjects treated with liraglutide or glimepiride

**DOI:** 10.1186/s12933-021-01431-2

**Published:** 2021-12-17

**Authors:** J. Jendle, T. Hyötyläinen, M. Orešič, T. Nyström

**Affiliations:** 1 Department of Medical Sciences, Campus USÖ, Örebro University, 70182 Örebro, Sweden; 2 School of Science and Technology, Örebro University, Örebro, Sweden; 3 Department of Clinical Science and Education, Karolinska Institutet, Södersjukhuset, Stockholm, Sweden

**Keywords:** Ceramide, Glimepiride, GLP-1 receptor agonist, Lipidomics, Liraglutide, Metabolomics, Myocardial infarction, Type 2 diabetes

## Abstract

**Background:**

Treatment with glucagon-like peptide-1 receptor agonists (GLP-1 RAs) leads to multiple metabolic changes, reduction in glucose levels and body weight are well established. In people with type 2 diabetes, GLP-1 RAs reduce the risk of cardiovascular (CV) disease and may also potentially represent a treatment for fatty liver disease. The mechanisms behind these effects are still not fully elucidated. The aim of the study was to investigate whether treatment with liraglutide is associated with favourable metabolic changes in cases of both CV disease and fatty liver disease.

**Methods:**

In a prespecified post-hoc analysis of a double-blind, placebo-controlled trial in 62 individuals with type 2 diabetes (GLP-1 RA liraglutide or glimepiride, both in combination with metformin), we evaluated the changes in plasma molecular lipids and polar metabolites after 18 weeks of treatment. The lipids and polar metabolites were measured by using ultra-high-performance liquid chromatography quadrupole time-of-flight mass spectrometry (UHPLC-QTOFMS).

**Results:**

In total, 340 lipids and other metabolites were identified, covering 14 lipid classes, bile acids, free fatty acids, amino acids and other polar metabolites. We observed more significant changes in the metabolome following liraglutide treatment compared to with glimepiride, particularly as regards decreased levels of cholesterol esters hexocyl-ceramides, lysophosphatidylcholines, sphingolipids and phosphatidylcholines with alkyl ether structure. In the liraglutide-treated group, lipids were reduced by approximately 15% from baseline, compared to a 10% decrease in the glimepiride group. At the pathway level, the liraglutide treatment was associated with lipid, bile acid as well as glucose metabolism, while glimepiride treatment was associated with tryptophan metabolism, carbohydrate metabolism, and glycerophospholipid metabolism.

**Conclusions:**

Compared with glimepiride, liraglutide treatment led to greater changes in the circulating metabolome, particularly regarding lipid metabolism involving sphingolipids, including ceramides. Our findings are hypothesis-generating and shed light on the underlying biological mechanisms of the CV benefits observed with GLP-1 RAs in outcome studies. Further studies investigating the role of GLP-1 RAs on ceramides and CV disease including fatty liver disease are warranted.

*Trial registration:* NCT01425580

**Supplementary Information:**

The online version contains supplementary material available at 10.1186/s12933-021-01431-2.

## Introduction

In type 2 diabetes (T2D), insulin resistance and increasing adiposity result in increased levels of free fatty acids (FFAs) which lead to fat storage in the liver and in the heart [1]. In the liver, this may cause non-alcoholic fatty liver disease (NAFLD), and, in the heart cardiac dysfunction, i.e., heart failure or coronary artery disease [2, 3]. This supports the hypothesis that these disturbances are related to lipotoxic environment due to dysfunctional adipose tissues and therefore cardiometabolic consequences [2, 4].

Treatment with glucagon-like peptide-1 receptor agonists (GLP-1 RAs) lead to multiple metabolic changes, whereas reduction in glucose levels and body weight are well-established indications for the treatment of T2D and or obesity [5]. In the LEADER (Liraglutide Effect and Action in Diabetes) trial [6] the time-to-event analysis for the composite endpoint, i.e., the rate of the first occurrence of cardiovascular (CV) death, non-fatal myocardial infarction, or non-fatal stroke was significantly lower among individual with T2D treated with liraglutide, compared to placebo. It was also recently shown that ameliorative effects occur in non-alcoholic steatohepatitis (NASH) in patients treated with liraglutide [7].

Beside the reduction in weight and glucose levels of liraglutide treatment, there are several indirect actions reported regarding heart function and the vessel evoked by activation of GLP-1 receptors (GLP-1 R) [8, 9]. These actions may include alterations in the substrate of fatty acids and glucose delivered to the heart and to the liver, altering vascular redox state, which may be a target for GLP-1 RAs [10].

In this post-hoc study, following 18 weeks treatment of liraglutide vs. glimepiride, both in combination with metformin in subjects with T2D with subclinical heart failure, our aim was to gain a comprehensive view of metabolomic changes via the use of high-resolution mass spectrometry.

## Methods

### Trial design

A post-hoc analysis from an assessor-blinded and active-controlled, parallel-group trial in combination with metformin in subjects with T2D and subclinical heart failure identified as NCT01425580 (www.clinicaltrials.gov). The main trial has been published elsewhere [11–13]. Briefly, T2D patients on oral glucose lowering therapy and with a glycated haemoglobin A1c (HbA1c) of 45‐97 mmol/mol (6.3–11%) were eligible if they had not been previously treated with GLP‐1 RAs, dipeptidyl peptidase‐4 inhibitors, glitazones, insulin or glimepiride.

Patients were invited for echocardiographic screening, given the following inclusion criteria: left ventricle ejection fraction  ≤  50% or evidence of diastolic dysfunction. The major exclusion criteria were: type 1 diabetes, heart failure according to the New York Heart Association classification 3–4, past history of atrial fibrillation or flutter, presence of acute myocarditis or significant valvulopathies, severe heart conduction disturbances or ventricular tachyarrhythmia, unstable angina or myocardial infarction in the previous 8 weeks, uncontrolled hypertension, estimated glomerular filtration rate (eGFR)  < 30 mL/min, haemoglobin  < 90 g/L, BMI  > 40 kg/m^2^, severe gastrointestinal disease, history of acute or chronic pancreatitis, malign neoplasia, current drug or alcohol abuse and pregnancy.

Patients were randomized between receiving either liraglutide or glimepiride during an 18‐week treatment period. The initial dose of liraglutide was 0.6 mg subcutaneously (s.c.), with an up‐titration of 0.6 mg every week to a final dose of 1.8 mg per day. The initial dose of the comparator was 2 mg glimepiride with an up‐titration of 1 mg every week, reaching a final dose of 4 mg per day.

### Aims of the study

Explorative endpoints were changes between study groups regarding metabolite profiles, including those previously associated with NAFLD, insulin resistance, or T2D, after 18 weeks of treatment with liraglutide or glimepiride both in combination with metformin.

#### Metabolomic analyses

### Sample preparation

Plasma samples, collected [1] at baseline and [2] after 18 weeks of treatment with liraglutide  +  metformin or glimepiride  +  metformin, were immediately centrifuged and aliquotted into Eppendorf tubes and stored at − 80 °C until analysis. Two separate extraction methods were used, one for lipidomics and one for polar metabolites. All samples were randomized before sample preparation and analysis. Pooled quality control samples, in-house quality control samples as well as NIST 1950 reference serum and extracted blank samples were analyzed to control for technical variation.

For lipidomics, the samples were extracted using a modified version of the previously published Folch procedure [14]. In short, 10 µL of serum was extracted with 120 µL of CHCl3: MeOH (2:1, v/v) containing the internal standards (c  = 2.5 µg/mL; 1,2-diheptadecanoyl-sn-glycero-3-phosphoethanolamine [PE(17:0/17:0)], N-heptadecanoyl-D-erythro-sphingosylphosphorylcholine [SM(d18:1/17:0)], N-heptadecanoyl-D-erythro-sphingosine [Cer(d18:1/17:0)], 1,2-diheptadecanoyl-sn-glycero-3-phosphocholine [PC(17:0/17:0)], 1-heptadecanoyl-2-hydroxy-sn-glycero-3-phosphocholine [LPC(17:0)] and 1-palmitoyl-d31-2-oleoyl-sn-glycero-3-phosphocholine [PC(16:0/d31/18:1)], were purchased from Avanti Polar Lipids, Inc. (Alabaster, AL, USA), and, triheptadecanoylglycerol [TG(17:0/17:0/17:0)] was purchased from Larodan AB (Solna, Sweden). The samples were stored at − 80 °C until analysis. The relative standard deviation (RSD%) for pooled samples was on average 15.8% and for in-house QC samples 18.1%.

For polar metabolites, 40 µL of sample was extracted with 400 µL of cold MeOH/H2O containing the following internal standard mixture: valine-d8, glutamic acid-d5, succinic acid-d4, heptadecanoic acid, lactic acid-d3, citric acid-d4. 3-hydroxybutyric acid-d4, arginine-d7, tryptophan-d5, glutamine-d5, 1-D4-cholic acid, 1-D4-chenodeoxycholic acid, 1-D4-glucocholic acid-D4-glycochenodeoxycholic acid, 1-D4-glycolithocholic acid, 1-D4-glycoursocholic acid, 1-D4-lithocholic acid1-D4-taurocholic acid and 1-D4-ursocholic acid. After centrifugation, the extracts were evaporated with nitrogen and reconstituted in the mobile phase. The relative standard deviation (RSD%) for pooled samples was on average 22.0% and for in-house QC samples 27.1%.

### Instrumental analysis

Three methods were used for analysis of the samples. All methods used ultra-high-performance liquid chromatography quadrupole time-of-flight mass spectrometry (UHPLC-QTOFMS) with 1290 Infinity II UHPLC system from Agilent Technologies (Santa Clara, CA, USA).

For lipidomics, the analysis was done as described in McGlinchey et al*.* [15]. The analysis of semipolar compounds was done as described in [16] except using quadrupole time-of-flight mass spectrometry instead of triple quadrupole. The MS conditions were as follows: a dual jet stream electrospray (dual ESI) ion source was used, in negative ion mode. The capillary voltage and the nozzle voltage were kept at 4500 V and 1500 V, respectively. The N_2_ pressure was set at 21 psi, with the sheath gas flow as 11 L/min and temperature at 379 °C for the nebulizer. Analysis of highly-polar metabolites was done as described in [17].

MS data processing was performed using open-source software MZmine 2.53 [10]. Quantitation of the following metabolites (aspartic acid, glutamic acid, isoleucine, methionine, fumaric acid, malic acid, leucine, valine, glycerol-3-phosphate, alanine, threonine, 3-hydroxybutyric acid, isocitric acid, arachidonic acid, glutamine, lactic acid, linoleic acid, oleic acid, palmitic acid, stearic acid and lysine, beta-muricholic acid (b-MCA), cholic acid (CA), chenodeoxycholic acid (CDCA), deoxycholic acid (DCA), glycocholic acid (GCA), glycochenodeoxycholic acid (GCDCA), glycodeoxycholic acid (GDCA), glycohyocholic acid (GHCA), glycohyodeoxycholic acid (GHDCA), glycolitocholic acid (GLCA), glycoursodeoxycholic acid (GUDCA), hyocholic acid (HCA), hyodeoxycholic acid (HDCA), lithocholic acid (LCA), tauro-alpha/beta-muricholic acid (Ta,bMCA), taurocholic acid (TCA), taurochenodeoxycholic acid (TCDCA), Taurodeoxycholic acid (TDCA) taurodehydrocholic acid (TDHCA), trihydroxycholestanoic acid (THCA), taurohyodeoxycholic acid (THDCA), taurolitocholic acid (TLCA), tauroursodeoxycholic acid (TUDCA), tauro-omega-muricholic acid (TwMCA), ursodeoxycholic acid (UDCA), omega/alpha-muricholic acid (w/a-MCA) was done using authentic standards at six different concentrations, other metabolites were determined semi-quantitatively. For lipids, the quantitation wad one by using hexadecyl-2-(9Z-octadecenoyl)-sn-glycero-3-phosphocholine {PC[16:0e/18:1(9Z)]}, 1-(1Z-octadecenyl)-2-(9Z-octadecenoyl)-sn-glycero-3-phosphocholine {PC[18:0p/18:1(9Z)]}, 1-stearoyl-2-hydroxy-sn-glycero-3-phosphocholine [LPC(18:0)], 1-oleoyl-2-hydroxy-sn-glycero-3-phosphocholine [LPC(18:1)], 1-palmitoyl-2-oleoyl-sn-glycero-3-phosphoethanolamine [PE(16:0/18:1)], 1-(1Z-octadecenyl)-2-docosahexaenoyl-sn-glycero-3-phosphocholine [PC(18:0p/22:6)] and 1-stearoyl-2-linoleoyl-sn-glycerol [DG(18:0/18:2)], 1-(9Z-octadecenoyl)-sn-glycero-3-phosphoethanolamine [LPE(18:1)], N-(9Z-octadecenoyl)-sphinganine {Cer[d18:0/18:1(9Z)]}, 1-hexadecyl-2-(9Z-octadecenoyl)-sn-glycero-3-phosphoethanolamine [PE(16:0/18:1)] from Avanti Polar Lipids, 1-Palmitoyl-2-Hydroxy-sn-Glycero-3-Phosphatidylcholine [LPC(16:0)], 1,2,3 trihexadecanoalglycerol [TG(16:0/16:0/16:0)], 1,2,3-trioctadecanoylglycerol [TG(18:0/18:0/18:)] and 3β-hydroxy-5-cholestene-3-stearate [ChoE(18:0)], 3β-Hydroxy-5-cholestene-3-linoleate [ChoE(18:2)] from Larodan, Solna, Sweden).

#### Statistical analyses

Continuous data are summarized as mean  ±  SD and categorical data are presented as percentages. For metabolomics, compounds with  > 20% missing values were removed. For the remaining variables, imputation was used to fill missing values/below limit of detection (LOD), using a value equal to half of the minimum detected value. The data was subsequently log_2_ transformed prior to data analysis and adjusted for gender and myocardial infarction incidence, as these were the two parameters showing to have a significant impact on the metabolite levels. Other possibly confounding factors were also analyzed namely, gender, age, BMI and pharmacological treatment and other clinical parameters on metabolome using Spearman correlation. As the groups were well-balanced in relation to age and BMI, it was considered sufficient to use only adjustment for gender and myocardial infarction. The impact of treatment on metabolome was investigated and the impact of adjustment with gender and MI was further studied using a generalized linear model approach using IBM^®^’s SPSS^®^ Statistics, and Stata 14.2 software (StataCorp, College Station, TX, USA). For treatment effect, the fold change was calculated as a ratio of the metabolite concentration after treatment, divided by the concentration at baseline, both using pairwise fold changes as well as group level fold changes.

#### Pathway analysis

Pathway analyses using the metabolomic data were done by both mummichog as well as with Gene Set Enrichment Analysis (GSEA). Pathway overrepresentation analysis was performed using the MetaboAnalyst 4.0 web platform, using the Functional Analysis (MS Peaks) module and using both the Mummichog algorithm as well as the GSEA algorithm [18]. The *Homo* *sapiens* [KEGG] pathway library was used in the analyses. For the input data for pathway analysis, the complete high-resolution LC–MS spectral peak data acquired in negative ionization mode was used, with mass tolerance of 7 ppm for identification. The analytical method covers polar and semipolar metabolites, such as bile acids, amino acids, free fatty acids and their derivatives and polar lipids.

## Results

### Study cohort and the impact of clinical variables on metabolome

Baseline characteristics of the study population are shown in Table 1. Treatment groups were well-balanced except for triacylglycerols (TGs) and HbA1c which was higher in subjects randomized to liraglutide treatment (Table 1).

**Table 1:** Baseline characteristics of the study population

	Liraglutide *n* = 33	Glimepiride *n* = 29	p value
Age, years	60.8 (7.6)	63.0 (6.8)	0.240^a^
Male sex	24 (72.7)	21 (72.4)	1.000^b^
Diabetes duration, years	5 (1, 10)	1 (3, 7)	0.368^c^
Smoking	3 (9.1)	4 (13.8)	0.852^b^
BMI, kg/m^2^	30.5 (4.4)	29.0 (3.2)	0.152^a^
Body weight, kg	91.8 (15.9)	89.0 (9.9)	0.411^a^
Waist circumference, cm	109.0 (13.0)	106.3 (9.7)	0.366^a^
Mean systolic BP, mmHg	131.9 (14.0)	129.3 (10.9)	0.414^a^
Mean diastolic BP, mmHg	76.7 (7.9)	77.2 (7.9)	0.838^a^
eGFR, mL/min/1.72 m^2^	88.3 (15.0)	87.4 (13.1)	0.799^a^
Complications			
Hypertension	29 (87.9)	21 (72.4)	0.224^b^
Hyperlipidemia	25 (75.8)	23 (79.3)	0.980^b^
Myocardial infarction	10 (30.3)	11 (37.9)	0.714^b^
Stroke	1 (3)	2 (6.9)	0.902^b^
Proliferative retinopathy	1 (3)	1 (3.4)	1.000^b^
Treatment			
Antiplatelet therapy	11 (33.3)	12 (41.4)	0.696^b^
Anticoagulant treatment	3 (9.1)	1 (3.4)	0.714^b^
ACE inhibitors/ARB blockers	25 (75.8)	20 (69)	0.754^b^
Beta-blockers	14 (42.4)	13 (44.8)	1.000^b^
Calcium inhibitors	13 (39.4)	10 (34.5)	0.894^b^
Diuretics	11 (33.3)	6 (20.7)	0.408^b^
Statins	22 (66.7)	24 (82.8)	0.248^b^
Biochemical parameters			
HbA1c, mmol/mol	54 (50, 60)	50 (49, 54)	0.036^c^
Triglycerides, mmol/L	2.0 (1.4, 2.6)	1.5 (1.0, 2.2)	0.029^c^
Total cholesterol, mmol/L	4.4 (4.0, 6.0)	4.5 (3.7, 4.8)	0.370^c^
LDL-cholesterol, mmol/L	2.8 (1.2)	2.5 (1.0)	0.440^a^
HDL-cholesterol, mmol/L	1.1 (0.3)	1.2 (0.3)	0.417^a^

Quantitative data are mean (SD) or median (1st quartile, 3rd quartile), and categorical data are n (%)

*ARB* angiotensin receptor blockers; *BP* blood pressure; *eGFR* estimated glomerular filtration rate; *HbA1c* glycated hemoglobin A1c

^a^Student’s t test was used

^b^Doubled one-sided p value from Fisher’s exact test

^c^Mann–Whitney U test was used

**Fig. 1:**
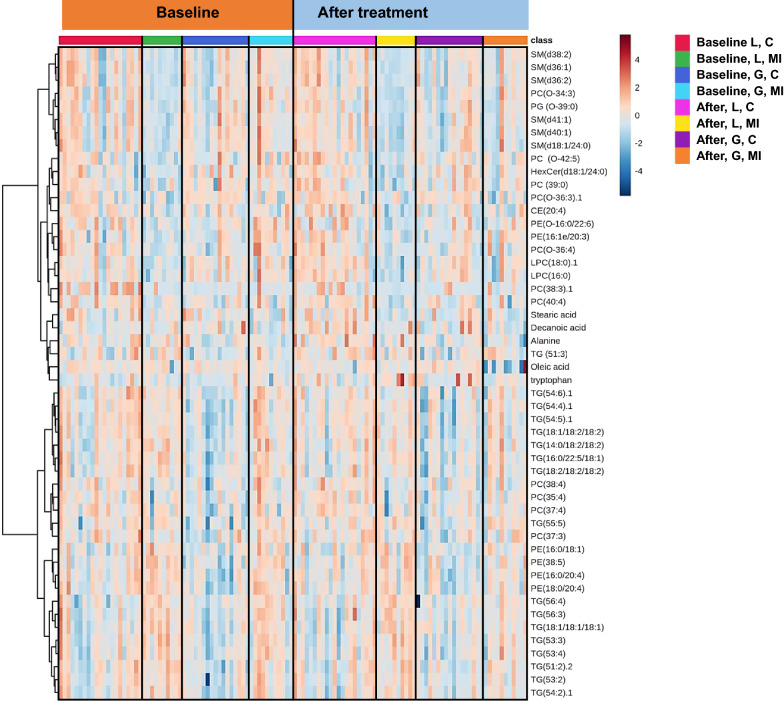
Heatmap depicting log_2_ transformed levels of metabolites at baseline and after treatment [glimepiride  +  metformin (G) vs. liraglutide  +  metformin (L)]. Groups were further divided into subjects with myocardial infarction (MI) or without MI (C, control)

At baseline, age, BMI, gender, and pharmacological treatment were associated with differences seen in several lipid classes (Additional file 1: Figure S1). Subjects with an earlier myocardial infarction were associated with higher levels of short, saturated TGs and other lipids when observing only subjects on statins (Fig. 1). However, the differences were relatively small, being on average 10% higher in those subjects that had had a myocardial infarction (Fig. 1). There were also some differences in metabolite profiles between the treatment groups at baseline, particularly among subjects with MI (Additional file 1: Figure S1).

In subjects with an earlier myocardial infarction there were specific changes in the metabolome at baseline, as compared with subjects without MI, with significantly higher levels of several TGs and lower levels of several phospholipids and free fatty acids (Additional file 1: Table S2).

### Treatment with liraglutide and glimepiride associates with distinct changes in the metabolome

Treatment with either liraglutide or glimepiride had a significant impact on the body weight with a reduction [− 3.7 vs. − 0.2 kg (− 5.5, − 1.4; p  = 0.001)] and the glimepiride group, respectively. In contrast, there were no significant changes in any of the clinical blood lipid levels: TGs [− 0.2 (0.4) vs. − 0.1 (0.8) mmol/L, p  = 0.492], LDL-cholesterol [− 0.1 (− 0.5, 0.1) vs. − 0.2 (− 0.5, 0.1) mmol/L p  = 0.994], and HDL cholesterol [0.1 (0.0, 0.2) vs. 0.0 (− 0.1, 0.1) mmol/L, p  = 0.38] between treatment groups, i.e., liraglutide and glimepiride groups, respectively.

**Fig. 2:**
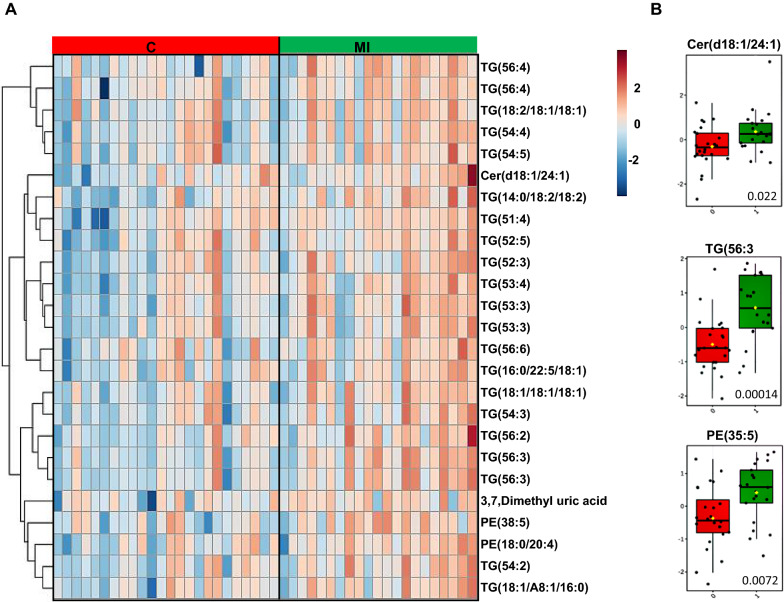
**A** Heatmap demonstrating differences in lipids, at baseline, between individuals with (n  = 21) or without myocardial infarction (MI) (n  = 41). **B** Selected examples of lipids, i.e., ceramide [Cer(d18:1/24:1)], triacylglycerol [TG(56:3)] and phosphatidylethanolamine [PE(35:5)], showing significant differences at baseline between groups with or without MI, with adjusted p values marked to the boxplots

In total, 340 lipids and other metabolites were identified, covering 14 lipid classes, bile acids, free fatty acids, amino acids, and other polar metabolites. We observed more significant changes from the baseline metabolome following liraglutide treatment compared versus glimepiride, particularly as regards decreased levels of cholesterol esters, hexocyl-ceramides, lysophosphatidylcholines, sphingomyelins and alkyl-ether-structured phosphatidylcholines. In the liraglutide-treated group, the lipids were reduced 15% from baseline level, compared with 10% change from baseline level in the glimepiride group. Each treatment caused significant changes in metabolic profile (Figs. 1,  2A, B). At the lipid class level, treatment with liraglutide decreased lipids overall, with significant decreases being observed in cholesterol esters (CEs), ceramides (Cers), sphingomyelins (SMs), lysophosphatidylcolines (LPCs) and alkyl_phosphatidylcholines (PC-Os) (Additional file 1: Table S3A). Glimepiride treatment resulted in decreases in several lipid classes as well, but the changes were less drastic, with significant decreases only in phosphatidylethanolamines (PEs) and those TGs with monounsaturated fatty acyls in their structure (TG-MUFAs) (Additional file 1: Table S3B).

**Fig. 3:**
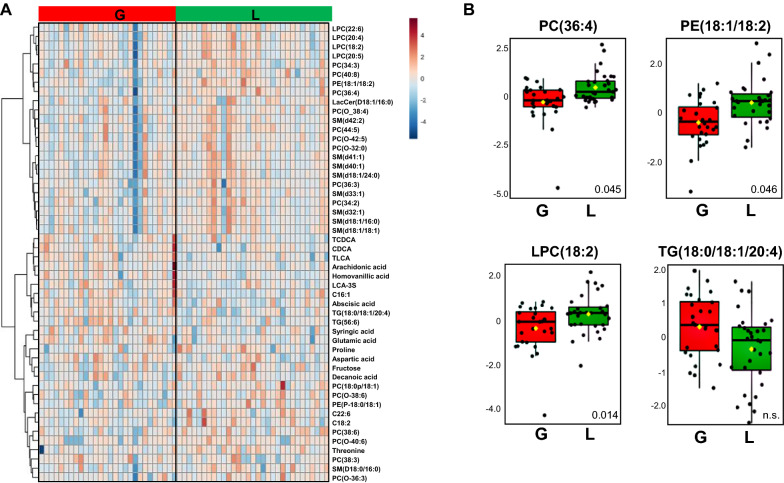
**A** Heatmap of fold changes between treated groups [glimepiride (G) vs. liraglutide (L)]. **B** Selected examples of lipids, i.e., phosphatidylcholine [PC(36:4)], phosphatidylethanolamines [PE(18:1/8:2)], lysophosphatidylcoline [LPC(18:2)] and triacylglycerol [TG(18:0/18:1/20:4)], showing significant difference between treated groups [glimepiride (G) vs. liraglutide (L)], with adjusted p values marked to the boxplots

At the level of individual metabolites, liraglutide treatmeant, on the other hand, altered the levels of multiple cholesterol esters, several phospholipids (PCs, LPCs, SMs), lactic acid and certain free fatty acids, amino acids and bile acids (Additional file 1: Table S4). Glimepiride treatment resulted in a significant impact on several free fatty acids, amino and bile acids and both TGs and several phospholipids (PE, SM) (Additional file 1: Table S5). The levels of the insulin resistance marker, 2-hydroxybutyric acid, [19] were increased in the glimepiride treatment group by comparison to baseline, while the levels of 3-hydroxybutyric acid, reported to be a cardioprotective marker [20], were decreased. The liraglutide treatment, on the other hand, caused downregulation of 3-hydroxybutyric acid. The impact of treatment showed significant differences with several metabolites showing difference between the two treatment groups (Fig. 3A, B; Additional file 1: Table S6). Particularly, several saturated fatty acids, some amino acids and TGs were lower in subjects treated with liraglutide while polyunsaturated FAs, valine, glycine and 3-hydroxybutyric acid were higher in this group.

We further investigated whether the change in BMI due to the treatments was associated with the changes in the metabolome, in both groups combined, and in the two treatment groups separately. The BMI changes were associated with specific lipids and metabolites, mainly phospholipids, but the association was not significant after FDR correction. However, two of the metabolites showing significant increase after glimepiride treatment (C16:1, methionine), showed a trend of positive correlation with the BMI change, being potentially related to the change in BMI.

### Treatment with liraglutide impacts multiple lipid-related metabolic pathways

**Fig. 4:**
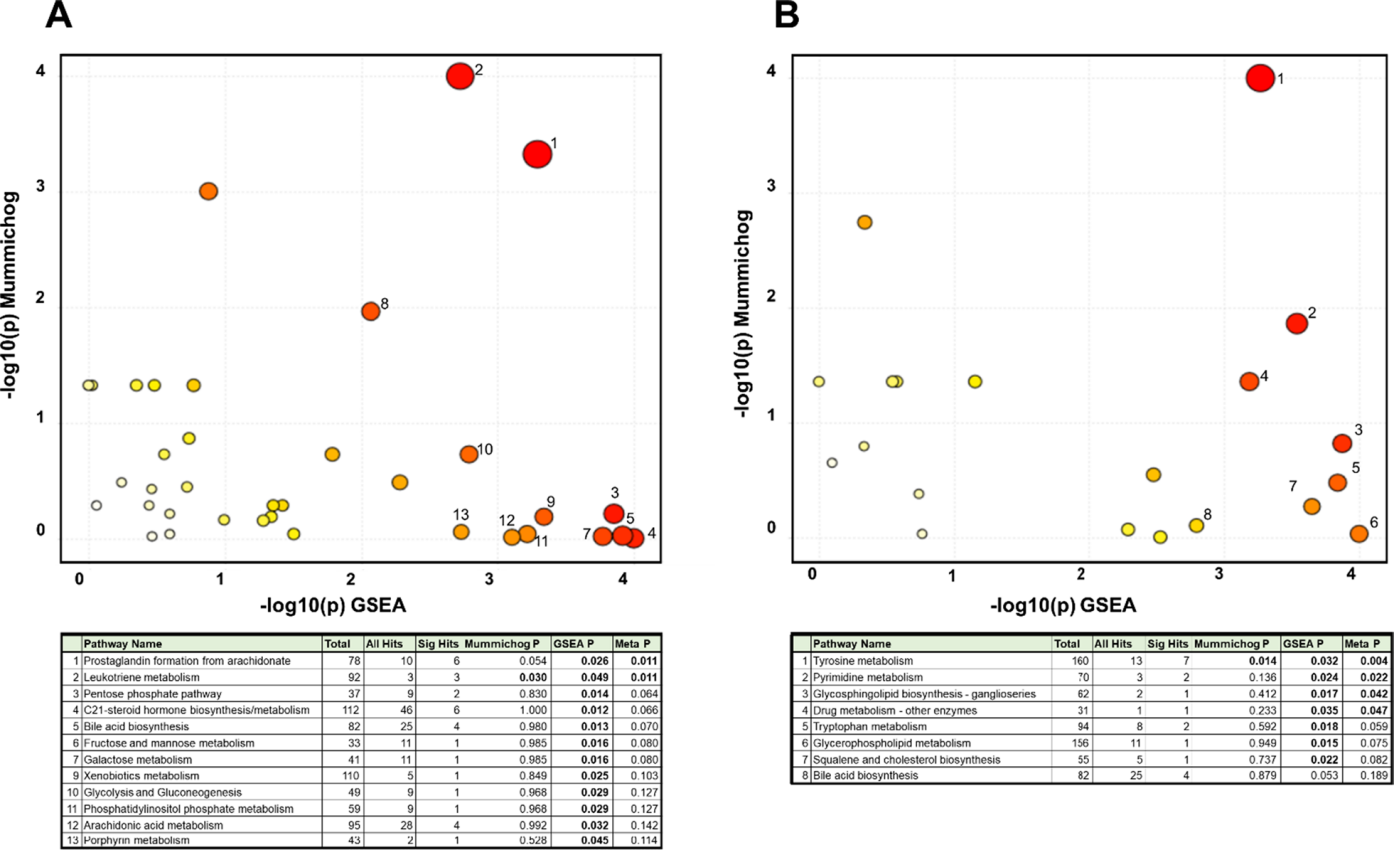
Pathway analysis performed on the metabolomic data were done by both mummichog as well as with Gene Set Enrichment Analysis (GSEA)

The pathway analyses showed that the two treatments resulted in impacts on different metabolic pathways. The treatment with liraglutide was associated with changes in several pathways, associated both with lipid, bile acid as well as glucose metabolism (Fig. 4). The most significant pathways impacted were related to arachidonic acid derived fatty acid metabolism (prostaglandin and leukotriene metabolism). Treatment with glimepiride was associated with fewer significant changes, mainly in tryptophan metabolism, carbohydrate metabolism as well as in glycerophospholipid metabolism.

## Discussion

This study aimed to gain a view of metabolic changes following the treatment with liraglutide and glimepiride in subjects with T2D with subclinical heart failure. By performing metabolomics analyses, we found that both treatments associated with distinct pharmacometabolomic profiles. The metabolic impact was greater in treatment with liraglutide particularly affecting multiple lipid-related pathways as compared to glimepiride.

Several clinical studies with the treatment of GLP-1 RAs shown improved lipid metabolism and amelioration of cardiometabolic risk factors [21]. The major effect of the treatment is known to be decreases in body weight and circulating glucose. Improvement of circulating lipid levels has been suggested to be secondary to these changes. Not only does treatment with GLP-1 RAs improve plasma lipids, but insulin and sulfonylurea, which both, in clinical studies, have shown a reduction in total and low-density lipoprotein cholesterol level with an increase in the ratio of high-density lipoprotein to low-density lipoprotein cholesterol [22].

Metabolomic studies in mice using nuclear magnetic resonance (NMR) spectroscopy, which is more restricted in terms of metabolomics coverage due to lower sensitivity than mass spectrometry, found that treatment with liraglutide alters β-oxidation in fatty acids also affects the microbiome, independently of other metabolic changes [23]. In agreement with this, recent human intervention studies using NMR spectroscopy (although with limited lipid coverage compared to mass spectrometry) were in line with our results, independent of other metabolic changes such as alterations in lipid and lipoprotein profile after liraglutide treatment compared to placebo [24, 25]. These results confirm that GLP-1 RAs (independently of changes in glycemic control and weight) alter lipid metabolism.

In the present study, there was a robust decrease in HbA1c, to a similar degree in both treated groups. However, in the liraglutide treated subjects, there was a significant reduction in body weight and waist circumference compared with subjects treated with glimepiride. Moreover, 2-hydroxybutyric acid, a marker of insulin resistance [19], was elevated after treatment with glimepiride but decreased on liraglutide treatment, possibly suggesting increased insulin resistance in the glimepiride group, and/or improved insulin sensitivity in the liraglutide group. Previous intervention studies indicate that treatment with liraglutide results in changes to lipid profiles, i.e., total cholesterol and LDL cholesterol levels both in subjects with and without T2D [24, 25]. Since these changes have been associated with weight loss it might simply be a consequence of other, unknown effects of the treatment [26]. In the present study, there were no changes in clinical lipid measurements, i.e., total cholesterol, LDL cholesterol, high-density lipoprotein (HDL) cholesterol or TGs between treatment groups. Despite this, treatment with liraglutide resulted in overall lowering of levels of various molecular lipids, as demonstrated by our lipidomic analyses. This may be due to an impact of GLP-1 on lipid absorption in the intestine, and its overall role in regulation of lipoprotein metabolism [27, 28]. Also, earlier studies of T2D subjects demonstrated that liraglutide treatment reduces postprandial lipidaemia, resulting in decreased liver fat, but no significant changes in the rate of hepatic de novo lipogenesis or markers of fat oxidation [25]. Even though no changes in the routinely measured plasma lipid levels were observed in any of the treated groups, there were distinct differences in metabolic changes between treatment groups. Specifically, liraglutide treatment resulted in more substantial decreases, in several lipid classes than glimepiride treatment did. Also, pathway analysis demonstrated that the liraglutide treatment had a more substantial impact on metabolic regulation than the glimepiride, particularly as regards to pathways related to lipidomic inflammatory mediators and bile acid metabolism.

Multiple sphingolipids, including glycoceramides, a class of bioactive sphingolipids, were found to be decreased in subjects treated with liraglutide [29]. Ceramides show evidence of being key mediators of lipotoxicity [30], and strong biomarkers of atherosclerosis [31]. Specifically, genetic variants in sphingolipid synthesis genes, particularly those involved in glycoceramide metabolism, have been associated with incidence of myocardial infarction [32]. Animal models have demonstrated that glycoceramides are markedly elevated in ischaemic heart disease [17]. Interestingly, in the present study, subjects with a former myocardial infarction were found having increased levels of glycoceramides at baseline, lending support to the notion of an association between ceramides and atherosclerosis. Recently, it was demonstrated that fat-secreted ceramides are modifiable regulators of vascular redox state with a direct impact on CV mortality in people with atherosclerosis and that liraglutide was a potential drug target to decrease the risk [10]. It was also recently found that accumulation of ceramides associates with de novo lipogenesis and insulin resistance leading to low-grade inflammation [33], which, in turn, is suggested to increase the risk of T2D and CV disease [34].

Increased accumulation of ceramides alongside accumulation of TGs and free fatty acids confer risk of NAFLD [35], which is a leading cause of liver-related morbidity and mortality [36]. NAFLD is considered as being related to metabolic syndrome, and is highly associated with peripheral and hepatic insulin resistance, obesity, T2D, dyslipidemia and CV disease [37]. A third of people with NAFLD are likely to progress to non-alcoholic steatohepatitis (NASH), defined as the presence of hepatic steatosis and lobular inflammation with hepatocyte injury with or without fibrosis [38]. In some cases, NASH can progress to liver cirrhosis and hepatocellular carcinoma (HCC) [39]. Since our data suggest that liraglutide treatment has a beneficial impact on lipids and the overall metabolome, including metabolic features of NAFLD, i.e., ceramides, liraglutide treatment may be considered as one potential drug with activity against NAFLD [40, 41].

Liraglutide treatment had only a minor impact on the levels of individual bile acids, amino acids and free fatty acids, while treatment with the glimepiride treatment resulted in increasing levels of multiple free fatty acids, bile acids and amino acids. According to our pathway analysis, liraglutide treatment was associated with bile acid pathways and arachidonic acid metabolism. Whilst it has been suggested that GLP‐1‐based therapies may have a role in biliary physiology, previous studies have not reported any major changes in fasting bile acid levels after treatment with liraglutide, with the secondary bile acids acid, deoxycholic acid (DCA), being an exception [42]. One of the explanations, for this finding, may be due to the co-treatment of metformin which as it is known to reduce serum bile acids and increase the intestinal bile acid pool, probably by decreasing ileal bile acid reuptake [43]. The arachidonic acid (AA) pathway, on the other hand, has been shown to play a key role in cardiovascular biology, particularly in relation to inflammatory processes [44, 45]. Both AA derivatives and bile acids can be classified as enzymatically-oxidized lipids. Enzymatic lipid oxidation is facilitated by a network of proteins that use polyunsaturated fatty acids (PUFA) such as arachidonic acid, or sterols as substrates, and specifically, cytochrome P450 (CYP) [46] account for many enzymes in both pathways, catalyzing the formation of both oxysterols as well as oxygenated PUFAs. Our results suggest that characterization of the metabolic pathways underlying the impact of the different treatment may help in the mechanistic understanding of the impact of the treatment, potentially also the adverse side-effects of the treatments. Arachidonic acid and its biologically-active fatty acid mediators are currently under consideration as novel preventive and therapeutic targets for cardiovascular diseases [44].

The strengths of this study include its double-blind, placebo-controlled nature, randomization and that we used three comprehensive parallel methods based on high-resolution mass spectrometry, to investigate metabolic changes in subjects with new compared to older antidiabetic drugs for the treatment of T2D.

There are also limitations of the study. We have no clinical data on insulin sensitivity; since liraglutide may play a role in reducing insulin resistance and endothelial dysfunction [47] the metabolic changes between groups may be explained by changes in insulin sensitivity evoked by liraglutide. We did not collect data regarding dietary habits of the study subjects. However, a recent study showed that overfeeding of carbohydrates increases levels of TG-SFAs, and decreases TG-PUFAs in LDL lipids, with no change in TG-MUFAs. Also, a high fat diet was found to have a similar, but stronger impact [48]. Since glimepiride administration did result in decreased TGs overall, and the difference between liraglutide and glimepiride treatments was mainly in TG-MUFA, it is unlikely that dietary factors had a major impact on the observed metabolic changes between the two treatments. Regarding body weight changes, the two groups were similar at the start of the trial but, after treatment, there was a significant reduction body weight (− 3.7 vs. − 0.2 kg) in the liraglutide group and the glimepiride groups respectively. However, the BMI alteration was not significantly associated with lipid changes brought on by treatment. Although the analytical coverage of the metabolites was good, we could not fully identify all metabolites detected. However, the pathway analysis tool does include the whole data and it also includes pathway to identify the unknown compounds, thus giving a representative view of the metabolic changes in the pathway level.

## Conclusion

In conclusion, our study demonstrated that treatment with liraglutide, more so than with glimepiride, leads to comprehensive changes in the circulating metabolome in individuals with T2D, particularly as regards lipid metabolism involving ceramides. As increased accumulation of ceramides highly associates with CV disease and NAFLD, further studies are warranted investigating the role of GLP-1 RAs in these conditions.

## Supplementary Information

**Additional file 1: Table S1.** Difference between treatment groups at baseline (adjusted for gender and myocardial infarction). **Table S2. **Difference between subjects with (n = 21) or without myocardial infarction (n = 41) at baseline, adjusted for gender and MI. **Table S3. **Changes in lipid class level in subjects with type 2 diabetes after treatment with **A** Liraglutide and **B** Glimepiride, adjusted for gender and myocardial infarction. **Table S4. **Metabolic changes after treatment with Liraglutide (adjusted for gender and myocardial infarction). **Table S5. **Metabolic changes after treatment with Glimepiride (adjusted for gender and myocardial infarction). **Table S6. **Difference after treatment (Liraglutide vs. Glimepiride, fold change treated/baseline), at group level, adjusted for gender and myocardial infarction. **Figure S1.** Spearman correlation between polar metabolites, lipid classes, age, gender, BMI, and pharmacological treatment, i.e., ASA/Clopidogrel, Warfarin/NOAC, ACEi/ARB, Calcium flow inhibitor and statins at baseline. *p < 0.05.

## Data Availability

Data from the clinical study are available upon request and an appropriate institutional collaboration agreement. These data are not available to access in a repository owing to concern that the identity of patients might be revealed inadvertently.
