# Supplementary material for: Pharmacometabolomic profiles in type 2 diabetic subjects treated with liraglutide or glimepiride
Source: Cardiovasc Diabetol. 2021 Dec 17;20:237. doi: 10.1186/s12933-021-01431-2 (PMC8684205; doi:10.1186/s12933-021-01431-2)
Supplement: Supplementary file 1 — Additional file 1: Table S1. Difference between treatment groups at baseline (adjusted for gender and myocardial infarction). Table S2. Difference between subjects with (n = 21) or without myocardial infarction (n = 41) at baseline, adjusted for gender and MI. Table S3. Changes in lipid class level in subjects with type 2 diabetes after treatment with A Liraglutide and B Glimepiride, adjusted for gender and myocardial infarction. Table S4. Metabolic changes after treatment with Liraglutide (adjusted for gender and myocardial infarction). Table S5. Metabolic changes after treatment with Glimepiride (adjusted for gender and myocardial infarction). Table S6. Difference after treatment (Liraglutide vs. Glimepiride, fold change treated/baseline), at group level, adjusted for gender and myocardial infarction. Figure S1. Spearman correlation between polar metabolites, lipid classes, age, gender, BMI, and pharmacological treatment, i.e., ASA/Clopidogrel, Warfarin/NOAC, ACEi/ARB, Calcium flow inhibitor and statins at baseline. *p < 0.05. [file 12933_2021_1431_MOESM1_ESM.docx]

**Supplementary material**

**Pharmacometabolomic profiles in type 2 diabetic subjects treated with liraglutide or glimepiride**

**Jendle J^1^, Hyötyläinen T^2^, Orešič M^1^, Nyström T^3^.**

1. Dept of Medical Sciences, Örebro University, Örebro, Sweden.
2. School of Science and Technology, Örebro University, Örebro, Sweden.
3. Dept of Clinical Science and Education. Karolinska Institutet. Södersjukhuset. Stockholm, Sweden

Corresponding author: Prof Johan Jendle, Dept of Medical Sciences, Campus USÖ, Örebro University, SE-70182 Örebro, Sweden. johan.jendle@oru.se

**Table S1.** Difference between treatment groups at baseline (adjusted for gender and myocardial infarction).

| **Metabolite** | **Mean Square** | **F** | **p** | **Partial Eta Squared** | **FOLD** |
| --- | --- | --- | --- | --- | --- |
| PE(38:5) | 2.18 | 6.64 | 0.0007 | 0.2850 | 0.81 |
| PE(16:0/20:4) | 2.13 | 6.44 | 0.0009 | 0.2787 | 0.84 |
| SM(39:2) | 2.50 | 5.64 | 0.0021 | 0.2527 | 1.08 |
| Glycochenodeoxycholicacid-3-O-sulfate | 2.88 | 5.21 | 0.0031 | 0.2212 | 0.84 |
| TG(53:3) | 1.20 | 5.06 | 0.0036 | 0.2162 | 0.94 |
| TG(54:4) | 1.51 | 4.89 | 0.0044 | 0.2106 | 0.80 |
| TG(18:1/18:2/18:2) | 1.65 | 4.82 | 0.0048 | 0.2080 | 0.67 |
| TG(14:0/18:2/18:2) | 12.36 | 4.79 | 0.0049 | 0.2072 | 0.58 |
| PC(O-36:3) | 8.61 | 4.74 | 0.0055 | 0.2213 | 1.42 |
| PE(18:0/20:4) | 1.32 | 4.71 | 0.0057 | 0.2203 | 0.87 |
| SM(d41:2) | 0.69 | 4.60 | 0.0064 | 0.2161 | 1.02 |
| TG(51:2) | 1.85 | 4.48 | 0.0069 | 0.1964 | 0.72 |
| TG(56:3) | 2.03 | 4.37 | 0.0079 | 0.1924 | 0.95 |
| TG(54:5) | 0.93 | 4.14 | 0.0102 | 0.1843 | 0.86 |
| SM (37:1) / (18:1/19:0) | 1.07 | 3.87 | 0.0145 | 0.1885 | 1.00 |
| TG(16:0/22:5/18:1) or TG(20:4/18:1/18:1) | 1.64 | 3.82 | 0.0148 | 0.1723 | 0.69 |
| TG(18:0/18:1/20:4) | 1.53 | 3.80 | 0.0150 | 0.1718 | 0.71 |
| TG (48:4) | 1.02 | 3.71 | 0.0168 | 0.1682 | 1.08 |
| PE(16:0/22:6) | 1.55 | 3.66 | 0.0183 | 0.1802 | 0.81 |
| LPC(18:2) | 1.37 | 3.66 | 0.0184 | 0.1800 | 1.44 |
| TG(53:2) | 4.19 | 3.62 | 0.0186 | 0.1648 | 0.52 |
| TG(54:2) | 0.99 | 3.61 | 0.0188 | 0.1645 | 0.80 |
| PI(38:3) | 0.68 | 3.59 | 0.0199 | 0.1773 | 0.95 |
| SM(d41:1) | 0.60 | 3.51 | 0.0219 | 0.1738 | 1.08 |
| TG(54:6) | 1.29 | 3.48 | 0.0219 | 0.1594 | 0.73 |
| TG(53:4) | 1.11 | 3.46 | 0.0224 | 0.1587 | 0.85 |
| TG (56:3) | 1.57 | 3.41 | 0.0236 | 0.1570 | 1.11 |
| Threonine | 0.05 | 3.30 | 0.0269 | 0.1526 | 1.06 |
| TG(18:1/18:1/18:1) | 0.63 | 3.17 | 0.0315 | 0.1472 | 0.97 |
| GCDCA | 0.00 | 3.14 | 0.0325 | 0.1462 | 1.00 |
| TG(56:6) | 0.64 | 3.14 | 0.0325 | 0.1462 | 0.79 |
| TG(51:3) | 0.99 | 3.13 | 0.0327 | 0.1460 | 0.85 |
| SM(d36:2) | 0.45 | 3.14 | 0.0335 | 0.1584 | 1.04 |
| PC(38:3) | 8.62 | 3.05 | 0.0369 | 0.1547 | 0.85 |
| PG (O-39:0 | 0.58 | 3.00 | 0.0390 | 0.1527 | 1.10 |
| LPC(20:5) | 1.93 | 2.99 | 0.0395 | 0.1522 | 1.14 |
| TG (53:3) | 0.62 | 2.92 | 0.0420 | 0.1374 | 0.91 |
| TG (51:3) | 1.65 | 2.92 | 0.0420 | 0.1374 | 0.77 |
| TG(18:1/18:1/16:0) | 0.26 | 2.91 | 0.0427 | 0.1369 | 0.93 |
| TG(51:4) | 4.18 | 2.90 | 0.0431 | 0.1365 | 0.81 |
| TG(51:2) | 1.21 | 2.86 | 0.0450 | 0.1350 | 0.73 |
| TG(48:3) | 6.19 | 2.83 | 0.0468 | 0.1337 | 0.54 |
| TG(56:4) | 1.91 | 2.82 | 0.0473 | 0.1333 | 0.89 |
| PE(16:0/18:1) | 1.82 | 2.84 | 0.0474 | 0.1454 | 0.87 |
| SM(d18:1/16:0) | 0.37 | 2.81 | 0.0486 | 0.1445 | 1.10 |

**Table S2.** Difference between subjects with (n=21) or without myocardial infarction (n=41) at baseline, adjusted for gender and MI.

| **Compound** | **df** | **Mean Square** | **F** | **p** | **Partial Eta Squared** | **Fold Change (MI vs no MI)** |
| --- | --- | --- | --- | --- | --- | --- |
| TG(56:3) | 3 | 2.07 | 8.67 | 0.0001 | 0.39 | 1.58 |
| TG(56:3) | 3 | 1.80 | 7.21 | 0.0005 | 0.35 | 1.54 |
| Lactic acid | 3 | 7.61 | 7.00 | 0.0007 | 0.34 | 0.49 |
| Decanoic acid | 3 | 3.10 | 6.12 | 0.0015 | 0.31 | 0.81 |
| TG(14:0/18:2/18:2) | 3 | 13.37 | 5.90 | 0.0019 | 0.30 | 1.52 |
| TG(56:2) | 3 | 5.08 | 5.44 | 0.0030 | 0.28 | 2.05 |
| TG(18:1/18:1/18:1) | 3 | 0.62 | 5.38 | 0.0032 | 0.28 | 1.24 |
| Stearic acid | 3 | 1.10 | 5.37 | 0.0033 | 0.28 | 0.86 |
| TG(48:4) | 3 | 1.14 | 5.17 | 0.0040 | 0.27 | 0.98 |
| 2-hydroxybutyric acid | 3 | 5.30 | 5.15 | 0.0041 | 0.27 | 0.60 |
| TG(54:2) | 3 | 1.05 | 5.14 | 0.0041 | 0.27 | 1.24 |
| PC(32:1) | 3 | 2.98 | 5.20 | 0.0044 | 0.30 | 1.03 |
| PC(32:2) | 3 | 2.42 | 5.19 | 0.0044 | 0.30 | 0.96 |
| TG(48:3) | 3 | 9.22 | 4.78 | 0.0060 | 0.26 | 1.56 |
| Palmitic acid | 3 | 3.34 | 4.74 | 0.0063 | 0.26 | 0.81 |
| Glycochenodeoxycholic acid-3-O-sulfate* | 3 | 2.44 | 4.72 | 0.0064 | 0.26 | 0.98 |
| TG(14:0/18:2/18:2) | 3 | 5.34 | 4.60 | 0.0073 | 0.25 | 2.86 |
| PC(40:4) | 3 | 1.16 | 4.63 | 0.0077 | 0.28 | 0.88 |
| PC(O-36:3) | 3 | 8.12 | 4.62 | 0.0078 | 0.28 | 0.73 |
| TG(50:3) | 3 | 1.88 | 4.42 | 0.0088 | 0.24 | 1.18 |
| Succinic acid | 3 | 0.60 | 4.24 | 0.0107 | 0.24 | 0.95 |
| TG(54:3) | 3 | 0.42 | 4.23 | 0.0108 | 0.24 | 1.20 |
| TG(14:0/18:1/18:1) | 3 | 1.67 | 4.08 | 0.0126 | 0.23 | 1.19 |
| TG(53:3) | 3 | 0.77 | 3.99 | 0.0139 | 0.23 | 1.36 |
| CE(18:2) | 3 | 0.10 | 4.03 | 0.0143 | 0.25 | 0.99 |
| PC(O-36:3) | 3 | 6.49 | 4.02 | 0.0144 | 0.25 | 0.77 |
| PE(16:0/18:1) | 3 | 2.20 | 3.94 | 0.0157 | 0.25 | 1.34 |
| TG(18:1/12:0/18:1) | 3 | 4.86 | 3.87 | 0.0158 | 0.22 | 1.23 |
| Valine | 3 | 0.27 | 3.87 | 0.0158 | 0.22 | 1.06 |
| TG(18:1/18:1/16:0) | 3 | 0.29 | 3.85 | 0.0162 | 0.22 | 1.14 |
| TG(16:0/18:2/18:3) | 3 | 4.93 | 3.85 | 0.0163 | 0.22 | 1.57 |
| Oleic acid | 3 | 0.74 | 3.80 | 0.0171 | 0.22 | 0.93 |
| TG(46:2) | 3 | 10.57 | 3.79 | 0.0174 | 0.22 | 1.58 |
| SM (39:2) | 3 | 1.77 | 3.85 | 0.0174 | 0.24 | 0.83 |
| TG(53:4) | 3 | 0.92 | 3.78 | 0.0175 | 0.22 | 1.34 |
| PE(16:0/20:4) | 3 | 1.05 | 3.82 | 0.0180 | 0.24 | 1.22 |
| LPC(20:4) | 3 | 1.02 | 3.73 | 0.0196 | 0.24 | 1.16 |
| TG(51:4) | 3 | 4.86 | 3.65 | 0.0202 | 0.21 | 1.43 |
| TG(52:5) | 3 | 0.93 | 3.58 | 0.0218 | 0.21 | 1.23 |
| SM(d41:2) | 3 | 0.47 | 3.62 | 0.0220 | 0.23 | 0.97 |
| TG(14:0/16:0/18:1) | 3 | 5.34 | 3.52 | 0.0233 | 0.20 | 1.18 |
| PC(40:5) | 3 | 1.19 | 3.57 | 0.0233 | 0.23 | 0.92 |
| TG(56:4) | 3 | 1.55 | 3.50 | 0.0239 | 0.20 | 1.53 |
| TG(16:1/18:1/12:0) | 3 | 9.40 | 3.48 | 0.0243 | 0.20 | 1.36 |
| PE(16:0/22:6) | 3 | 1.15 | 3.46 | 0.0262 | 0.22 | 1.20 |
| TG(50:1) | 3 | 2.29 | 3.39 | 0.0268 | 0.20 | 1.32 |
| TG(16:0/16:0/16:0) | 3 | 10.91 | 3.39 | 0.0269 | 0.20 | 1.25 |
| TG(16:0/18:0/18:1) | 3 | 4.16 | 3.34 | 0.0283 | 0.20 | 1.30 |
| TG(50:3) | 3 | 1.08 | 3.34 | 0.0284 | 0.20 | 1.14 |
| TG(50:2) | 3 | 1.12 | 3.31 | 0.0292 | 0.20 | 1.17 |
| TG(51:3) | 3 | 1.00 | 3.31 | 0.0294 | 0.19 | 1.24 |
| Alanine | 3 | 1.40 | 3.30 | 0.0295 | 0.19 | 0.93 |
| TG(54:7) | 3 | 4.04 | 3.29 | 0.0299 | 0.19 | 1.38 |
| TG(46:0 | 3 | 7.42 | 3.28 | 0.0302 | 0.19 | 1.24 |
| PC(35:3) | 3 | 1.42 | 3.30 | 0.0312 | 0.22 | 0.93 |
| TG(52:2) | 3 | 0.44 | 3.22 | 0.0324 | 0.19 | 1.17 |
| PC(30:0) | 3 | 2.23 | 3.26 | 0.0325 | 0.21 | 1.09 |
| TG(51:2) | 3 | 1.45 | 3.20 | 0.0331 | 0.19 | 1.22 |
| PE(18:1/18:2) | 3 | 2.29 | 3.22 | 0.0340 | 0.21 | 1.47 |
| TG(54:2) | 3 | 0.74 | 3.15 | 0.0351 | 0.19 | 1.29 |
| TG(53:2) | 3 | 2.24 | 3.14 | 0.0356 | 0.19 | 1.41 |
| TG (55:1) | 3 | 1.58 | 3.13 | 0.0359 | 0.19 | 1.15 |
| TG(16:0/22:5/18:1) or TG(20:4/18:1/18:1) | 3 | 0.98 | 3.09 | 0.0373 | 0.18 | 1.29 |
| 3,7-dimethyluric acid | 3 | 9.93 | 3.07 | 0.0383 | 0.18 | 0.95 |
| TG(18:1/18:2/18:2) | 3 | 0.88 | 3.04 | 0.0394 | 0.18 | 1.20 |
| Glutamic acid | 3 | 2.29 | 2.99 | 0.0421 | 0.18 | 1.19 |
| Prostaglandin A2 | 3 | 11.94 | 2.96 | 0.0433 | 0.18 | 1.68 |
| TG(56:4) | 3 | 1.53 | 2.95 | 0.0440 | 0.18 | 1.40 |
| TG(54:6) | 3 | 1.14 | 2.89 | 0.0469 | 0.17 | 0.96 |
| TG(16:0/18:2/18:2) | 3 | 0.76 | 2.85 | 0.0490 | 0.17 | 1.22 |
| SM(d39:1) | 3 | 0.43 | 2.88 | 0.0494 | 0.19 | 0.97 |

**Table S3.** Changes in lipid class level in subjects with type 2 diabetes after treatment with **A)** Liraglutide and **B**) Glimepiride, adjusted for gender and myocardial infarction.

**A)**

| **Lipid class** | **Mean Square** | **F** | **p** | **Partial Eta Squared** | **FOLD** |
| --- | --- | --- | --- | --- | --- |
| CE | 0.323 | 6.671 | **0.0002** | 0.319 | **0.89** |
| Cer | 0.183 | 0.897 | 0.4721 | 0.059 | 0.89 |
| DG | 0.651 | 2.034 | 0.1017 | 0.125 | 0.88 |
| Lac/HexCer | 0.534 | 4.715 | **0.0023** | 0.249 | **0.87** |
| LPC | 0.540 | 2.680 | **0.0406** | 0.158 | **0.87** |
| PC | 0.681 | 1.724 | 0.1574 | 0.108 | 0.87 |
| PC_O | 0.489 | 2.945 | **0.0278** | 0.171 | **0.87** |
| PE | 0.077 | 0.301 | 0.8762 | 0.021 | 0.87 |
| PE_O | 0.551 | 2.181 | 0.0826 | 0.133 | 0.87 |
| PI | 0.240 | 1.003 | 0.4134 | 0.066 | 0.88 |
| SM | 0.636 | 5.479 | **0.0008** | 0.278 | **0.88** |
| TG_SFA | 0.644 | 0.927 | 0.4547 | 0.061 | 0.87 |
| TG_MUFA | 0.143 | 0.253 | 0.9067 | 0.017 | 0.88 |
| TG_PUFA | 0.179 | 0.273 | 0.8942 | 0.019 | 0.88 |
| TG | 0.382 | 0.687 | 0.6037 | 0.046 | 0.88 |

**B)**

| **Lipid class** | **Mean Square** | **F** | **p** | **Partial Eta Squared** | **FOLD** |
| --- | --- | --- | --- | --- | --- |
| CE | 0.078 | 1.275 | 0.292 | 0.091 | 0.92 |
| Cer | 0.156 | 0.550 | 0.700 | 0.041 | 0.92 |
| DG | 0.406 | 1.401 | 0.247 | 0.099 | 0.92 |
| Lac/HexCer | 0.069 | 0.515 | 0.725 | 0.039 | 0.93 |
| LPC | 0.197 | 0.867 | 0.490 | 0.064 | 0.92 |
| PC | 0.401 | 0.837 | 0.508 | 0.062 | 0.91 |
| PC_O | 0.103 | 0.563 | 0.691 | 0.042 | 0.90 |
| PE | 0.829 | 5.830 | **0.001** | 0.314 | **0.90** |
| PE_O | 0.301 | 1.413 | 0.243 | 0.100 | 0.91 |
| PI | 0.339 | 1.724 | 0.159 | 0.119 | 0.90 |
| SM | 0.159 | 1.426 | 0.239 | 0.101 | 0.90 |
| TG_SFA | 0.872 | 1.546 | 0.203 | 0.108 | 0.91 |
| TG_MUFA | 1.362 | 3.245 | **0.019** | 0.203 | **0.91** |
| TG_PUFA | 1.219 | 2.400 | 0.062 | 0.158 | 0.90 |
| TG | 1.026 | 2.491 | 0.055 | 0.163 | 0.88 |

**Table S4.** Metabolic changes after treatment with Liraglutide (adjusted for gender and myocardial infarction).

| **Dependent Variable** | **Mean Square** | **F** | **p** | **Partial Eta Squared** | **FOLD** |
| --- | --- | --- | --- | --- | --- |
| SM(40:2) | 1.924 | 9.125 | 0.0001 | 0.341 | 1.031 |
| LPC(18:0) | 1.425 | 8.601 | 0.0001 | 0.327 | 1.163 |
| SM(d36:1) | 1.199 | 8.447 | 0.0001 | 0.323 | 0.996 |
| SM (37:1) | 2.834 | 8.273 | 0.0001 | 0.319 | 1.073 |
| SM(d41:2) | 1.091 | 8.084 | 0.0002 | 0.314 | 0.966 |
| PG (O-39:0) | 1.161 | 8.010 | 0.0002 | 0.312 | 0.935 |
| SM(d36:2) | 0.989 | 7.995 | 0.0002 | 0.312 | 1.009 |
| PC(O-34:3) | 0.928 | 7.551 | 0.0003 | 0.299 | 0.981 |
| CE(20:4) | 0.523 | 7.379 | 0.0003 | 0.295 | 1.001 |
| SM(d41:1) | 1.100 | 7.237 | 0.0004 | 0.291 | 1.002 |
| PC(O-40:5) | 1.418 | 7.231 | 0.0004 | 0.290 | 0.950 |
| TG (48:4) | 1.529 | 6.887 | 0.0005 | 0.263 | 0.956 |
| SM(d33:1) | 0.882 | 6.966 | 0.0005 | 0.283 | 1.010 |
| PC (42:8) | 2.402 | 6.857 | 0.0005 | 0.280 | 0.931 |
| PC(40:4) | 1.614 | 6.813 | 0.0006 | 0.278 | 1.049 |
| SM(d38:2) | 1.052 | 6.771 | 0.0006 | 0.277 | 0.994 |
| SM (d39:1) | 0.788 | 6.161 | 0.0011 | 0.259 | 0.963 |
| SM(d40:1) | 1.032 | 6.007 | 0.0013 | 0.254 | 0.955 |
| SM (d42:3) | 0.891 | 5.818 | 0.0016 | 0.248 | 0.991 |
| PC (O-42:5) | 1.661 | 5.764 | 0.0017 | 0.246 | 1.130 |
| SM(d18:1/16:0) | 0.680 | 5.734 | 0.0018 | 0.245 | 1.031 |
| PC(O-36:3) | 9.502 | 5.727 | 0.0018 | 0.245 | 1.226 |
| PG (O-41:0) | 1.589 | 5.650 | 0.0020 | 0.242 | 0.980 |
| PI (44:4) | 1.300 | 5.196 | 0.0032 | 0.227 | 0.989 |
| Prostaglandin A2* | 36.022 | 4.824 | 0.0046 | 0.200 | 0.849 |
| PC(O-36:4) | 0.876 | 4.704 | 0.0055 | 0.210 | 0.989 |
| LacCer(d18:1/16:0) | 0.831 | 4.688 | 0.0056 | 0.210 | 1.075 |
| PC(O-38:4) | 0.726 | 4.590 | 0.0063 | 0.206 | 1.034 |
| Threonine | 2.502 | 4.531 | 0.0064 | 0.190 | 1.040 |
| PI(38:3) | 0.918 | 4.574 | 0.0064 | 0.206 | 0.895 |
| CE(16:0) | 0.205 | 4.564 | 0.0064 | 0.205 | 0.963 |
| PC(38:3) | 13.172 | 4.562 | 0.0065 | 0.205 | 0.932 |
| PE(O-16:0/22:6) | 5.478 | 4.560 | 0.0065 | 0.205 | 0.778 |
| HexCer(d18:1/24:0) | 0.613 | 4.538 | 0.0066 | 0.204 | 1.010 |
| PC(O-32:0) | 0.842 | 4.472 | 0.0072 | 0.202 | 1.049 |
| Arachidonic acid | 1.951 | 4.328 | 0.0080 | 0.183 | 0.808 |
| SM(d42:2) | 0.957 | 4.359 | 0.0081 | 0.198 | 0.999 |
| PC(40:5) | 1.512 | 4.327 | 0.0084 | 0.197 | 1.006 |
| PC(O-38:5) | 0.659 | 4.316 | 0.0085 | 0.196 | 1.020 |
| SM(d18:0/16:0) | 0.912 | 4.253 | 0.0091 | 0.194 | 1.009 |
| PC(16:0e/18:1) | 0.668 | 4.182 | 0.0099 | 0.191 | 1.026 |
| CE(18:0) | 3.025 | 4.041 | 0.0116 | 0.186 | 1.109 |
| LPC(16:0) | 0.613 | 3.970 | 0.0126 | 0.183 | 1.100 |
| Taurolithocholic acid -3-O-sulfate* | 11.643 | 3.915 | 0.0129 | 0.168 | 0.638 |
| HDCA | 0.002 | 3.913 | 0.0130 | 0.168 | 1.000 |
| SM(39:2) | 0.842 | 3.909 | 0.0135 | 0.181 | 0.965 |
| PE(16:1e/20:3) | 1.606 | 3.857 | 0.0143 | 0.179 | 1.161 |
| PC(40:5) | 0.815 | 3.804 | 0.0152 | 0.177 | 1.002 |
| PE(O-38:5) | 0.835 | 3.728 | 0.0166 | 0.174 | 1.062 |
| CE(18:2) | 0.126 | 3.716 | 0.0169 | 0.174 | 0.995 |
| PC(36:2) | 1.295 | 3.641 | 0.0184 | 0.171 | 1.019 |
| PC (39:0) | 1.089 | 3.628 | 0.0186 | 0.170 | 1.015 |
| PE(P-16:0/20:4) | 0.806 | 3.564 | 0.0201 | 0.168 | 0.960 |
| TG(18:1/18:1/18:1) | 0.777 | 3.529 | 0.0203 | 0.154 | 0.992 |
| CE(20:5) | 1.065 | 3.546 | 0.0205 | 0.167 | 0.892 |
| PI (38:7) | 0.236 | 3.533 | 0.0208 | 0.167 | 1.145 |
| TG(56:4) | 2.613 | 3.503 | 0.0209 | 0.153 | 1.150 |
| Tryptophan | 0.000 | 3.460 | 0.0220 | 0.152 | 1.006 |
| 2-Hydroxybutyric acid | 4.092 | 3.510 | 0.021 | 0.154 | 0.71 |
| PC(O-34:3) | 1.050 | 3.439 | 0.0232 | 0.163 | 1.053 |
| PC(O-38:5) | 0.699 | 3.403 | 0.0242 | 0.162 | 1.014 |
| PC(38:6) | 10.358 | 3.365 | 0.0253 | 0.160 | 1.052 |
| Valine | 0.667 | 3.324 | 0.0258 | 0.147 | 0.951 |
| Glycine | 1.207 | 3.186 | 0.0303 | 0.141 | 0.954 |
| SM(d32:1) | 0.471 | 3.177 | 0.0314 | 0.152 | 1.004 |
| TG(54:5) | 0.458 | 3.136 | 0.0322 | 0.140 | 1.031 |
| Chenodeoxycholic  acid-3-glucuronide* | 5.995 | 3.121 | 0.0328 | 0.139 | 0.961 |
| PC(38:4) | 0.508 | 3.129 | 0.0332 | 0.150 | 1.010 |
| PC(38:5) | 0.230 | 3.104 | 0.0342 | 0.149 | 0.958 |
| PC(O-36:5) | 0.651 | 3.101 | 0.0343 | 0.149 | 0.956 |
| C22:6 | 2.018 | 3.081 | 0.0344 | 0.137 | 0.835 |
| SM(d18:1/24:0) | 0.694 | 3.077 | 0.0353 | 0.148 | 0.950 |
| TG(53:3) | 0.852 | 3.037 | 0.0362 | 0.136 | 0.979 |
| Lactic acid | 0.920 | 3.021 | 0.0369 | 0.135 | 0.973 |
| PC(35:4) | 0.847 | 3.018 | 0.0378 | 0.146 | 1.056 |
| Stearic acid | 0.752 | 2.988 | 0.0383 | 0.134 | 0.963 |
| PC(40:8) | 1.305 | 2.991 | 0.0390 | 0.145 | 1.301 |
| PC(36:4) | 1.543 | 2.986 | 0.0393 | 0.145 | 1.180 |
| TG(56:3) | 1.949 | 2.944 | 0.0404 | 0.132 | 0.996 |
| PC(36:4) | 0.108 | 2.879 | 0.0445 | 0.140 | 0.992 |
| SM(d34:2) | 0.464 | 2.802 | 0.0487 | 0.137 | 1.038 |
| PC(O-38:4) | 0.916 | 2.798 | 0.0489 | 0.137 | 1.082 |

*putative identification

**Table S5.** Metabolic changes after treatment with Glimepiride (adjusted for gender and myocardial infarction).

| **Dependent Variable** | **Mean Square** | **F** | **p** | **Partial Eta Squared** | **FOLD** |
| --- | --- | --- | --- | --- | --- |
| 3-Hydroxybutyric acid | 12.721 | 6.921 | 0.0005 | 0.285 | 0.907 |
| Palmitic acid | 4.622 | 6.354 | 0.0009 | 0.268 | 0.984 |
| Oleic acid | 19.807 | 6.258 | 0.0010 | 0.265 | 1.110 |
| PE(16:0/18:1) | 2.419 | 5.534 | 0.0025 | 0.269 | 0.669 |
| Abscisic Acid | 11.772 | 5.247 | 0.0031 | 0.232 | 1.610 |
| PE(16:0/22:6) | 1.702 | 5.156 | 0.0038 | 0.256 | 0.900 |
| PE(16:0/20:4) | 1.837 | 5.055 | 0.0042 | 0.252 | 1.064 |
| TG(54:4) | 1.953 | 4.526 | 0.0068 | 0.207 | 0.989 |
| TG(55:5) | 3.256 | 4.526 | 0.0068 | 0.207 | 1.030 |
| PE(18:0/20:4) | 1.374 | 4.582 | 0.0070 | 0.234 | 0.971 |
| TG(53:3) | 0.827 | 4.488 | 0.0071 | 0.206 | 1.037 |
| Linoleic acid | 4.951 | 4.327 | 0.0085 | 0.200 | 0.965 |
| CA | 0.020 | 4.290 | 0.0089 | 0.198 | 1.010 |
| TG(56:3) | 1.269 | 4.273 | 0.0090 | 0.198 | 1.034 |
| TG(51:4) | 4.360 | 4.018 | 0.0120 | 0.188 | 0.939 |
| SM (37:1) | 1.293 | 4.059 | 0.0123 | 0.213 | 0.913 |
| TG(53:4) | 1.176 | 3.865 | 0.0143 | 0.182 | 1.038 |
| TG(54:7) | 6.247 | 3.843 | 0.0147 | 0.181 | 0.722 |
| Stearic acid | 1.008 | 3.719 | 0.0169 | 0.177 | 1.045 |
| PE(38:5) | 1.575 | 3.745 | 0.0174 | 0.200 | 0.949 |
| TG(50:5) | 6.445 | 3.644 | 0.0184 | 0.174 | 0.791 |
| Threonine | 0.130 | 3.613 | 0.0191 | 0.172 | 1.175 |
| TG (53:3) | 0.633 | 3.591 | 0.0196 | 0.172 | 1.090 |
| TG(52:6) | 6.927 | 3.581 | 0.0198 | 0.171 | 0.921 |
| TG(14:0/18:2/18:2) | 9.441 | 3.558 | 0.0203 | 0.170 | 1.083 |
| TG(18:2/18:2/18:2) | 3.009 | 3.477 | 0.0223 | 0.167 | 0.765 |
| GLCA | 33.308 | 3.461 | 0.0228 | 0.166 | 1.219 |
| TG(18:1/18:2/18:2) | 1.408 | 3.459 | 0.0228 | 0.166 | 0.962 |
| SM(d41:2) | 0.811 | 3.470 | 0.0237 | 0.188 | 0.950 |
| C16:1 | 6.069 | 3.390 | 0.0247 | 0.164 | 1.469 |
| 2-hydroxybutyric acid | 3.498 | 3.346 | 0.0260 | 0.162 | 1.138 |
| PE(18:0/22:6) | 2.427 | 3.354 | 0.0270 | 0.183 | 1.00 |
| TG(18:2/18:2/18:2) | 5.045 | 3.311 | 0.0270 | 0.160 | 0.768 |
| LCA-3S | 10.929 | 3.295 | 0.0276 | 0.160 | 1.210 |
| Octanoic acid | 0.665 | 3.235 | 0.0295 | 0.157 | 1.011 |
| PE(18:1/18:2) | 1.735 | 3.267 | 0.0298 | 0.179 | 0.811 |
| Methionine | 3.244 | 3.228 | 0.0298 | 0.157 | 1.094 |
| TG(53:5) | 3.700 | 3.223 | 0.0299 | 0.157 | 0.843 |
| PE(16:1e/20:3) | 1.032 | 3.235 | 0.0309 | 0.177 | 0.952 |
| C22:6 | 4.522 | 3.174 | 0.0317 | 0.155 | 1.090 |
| Chenodeoxycholic  acid-3-glucuronide | 9.503 | 3.173 | 0.0317 | 0.155 | 1.081 |
| TG(58:9) | 5.092 | 3.157 | 0.0323 | 0.154 | 0.674 |
| TG (56:3) | 0.997 | 3.148 | 0.0327 | 0.154 | 0.868 |
| TG (48:4) | 0.775 | 3.138 | 0.0331 | 0.153 | 0.962 |
| TCDCA | 11.379 | 3.121 | 0.0337 | 0.153 | 0.915 |
| C18:2 | 0.000 | 3.035 | 0.0373 | 0.149 | 1.000 |
| TG(16:0/18:2/18:3) | 4.667 | 3.016 | 0.0381 | 0.148 | 0.933 |
| Arachidonic acid | 3.746 | 2.998 | 0.0389 | 0.147 | 1.098 |
| Decanoic acid | 3.038 | 2.915 | 0.0428 | 0.144 | 1.038 |
| TG(51:3) | 0.871 | 2.914 | 0.0429 | 0.144 | 1.066 |
| SM(39:2) | 0.489 | 2.932 | 0.0436 | 0.164 | 0.940 |
| TG(14:0/18:2/18:2) | 4.052 | 2.893 | 0.0440 | 0.143 | 0.930 |
| Proline | 0.397 | 2.883 | 0.0445 | 0.143 | 1.046 |
| SM(40:2) | 0.766 | 2.892 | 0.0456 | 0.162 | 1.016 |
| SM(d33:1) | 0.348 | 2.882 | 0.0461 | 0.161 | 1.001 |
| TG(52:5) | 1.048 | 2.850 | 0.0462 | 0.141 | 0.949 |
| TG(54:5) | 0.792 | 2.846 | 0.0464 | 0.141 | 0.943 |
| TG(16:0/18:2/22:6) | 5.185 | 2.824 | 0.0477 | 0.140 | 0.703 |
| TG (49:3) | 3.076 | 2.814 | 0.0482 | 0.140 | 0.987 |
| LPC(20:4) | 0.833 | 2.836 | 0.0486 | 0.159 | 1.074 |
| SM (d42:3) | 0.452 | 2.821 | 0.0495 | 0.158 | 0.962 |
| Tryptophan | 0.000 | 2.786 | 0.0498 | 0.138 | 1.001 |

**Table S6.** Difference after treatment (Liraglutide *vs.* Glimepiride, fold change treated/baseline), at group level, adjusted for gender and myocardial infarction.

| **Metabolite** | **Mean Square** | **F** | **p** | **Partial Eta Squared** | **Fold (L vs G)** |
| --- | --- | --- | --- | --- | --- |
| PE(16:0/18:1) | 1.96 | 3.50 | 0.0224 | 0.18 | 0.59 |
| Decanoic acid | 3.09 | 3.05 | 0.0362 | 0.14 | 0.69 |
| TG(18:1/18:2/18:2) | 1.36 | 3.35 | 0.0255 | 0.15 | 0.72 |
| PC(40:4) | 1.08 | 3.20 | 0.0314 | 0.17 | 0.75 |
| Palmitic acid | 2.30 | 3.05 | 0.0361 | 0.14 | 0.80 |
| TG(53:3) | 0.72 | 3.02 | 0.0376 | 0.14 | 0.81 |
| Stearic acid | 1.04 | 4.21 | 0.0094 | 0.19 | 0.82 |
| Alanine | 2.41 | 5.04 | 0.0037 | 0.22 | 0.82 |
| SM (39:2) | 1.07 | 4.27 | 0.0095 | 0.21 | 0.84 |
| TG (48:4) | 0.74 | 3.34 | 0.0257 | 0.15 | 0.87 |
| TG(18:2/18:2/18:2) | 4.51 | 2.84 | 0.0461 | 0.13 | 0.89 |
| SM(37:1) | 0.89 | 3.69 | 0.0180 | 0.19 | 0.90 |
| SM(d41:1) | 0.49 | 3.08 | 0.0363 | 0.16 | 0.91 |
| TG(51:2) | 1.18 | 2.94 | 0.0411 | 0.14 | 0.91 |
| TG(55:5) | 1.93 | 4.02 | 0.0117 | 0.18 | 0.91 |
| Lactic acid | 1.34 | 3.46 | 0.0223 | 0.16 | 0.92 |
| Serine | 1.62 | 4.61 | 0.0060 | 0.20 | 0.93 |
| SM(d33:1) | 0.64 | 6.16 | 0.0012 | 0.28 | 1.06 |
| PG (O-39:0) | 0.57 | 3.94 | 0.0137 | 0.20 | 1.08 |
| Valine | 0.25 | 3.54 | 0.0205 | 0.16 | 1.08 |
| PG (O-41:0) | 1.05 | 4.49 | 0.0074 | 0.22 | 1.08 |
| Glycine | 1.98 | 3.92 | 0.0132 | 0.18 | 1.20 |
| C22:6 | 4.85 | 3.31 | 0.0265 | 0.15 | 1.22 |
| Linoleic acid | 0.75 | 4.09 | 0.0108 | 0.18 | 1.28 |
| 3-Hydroxybutyric acid | 7.09 | 3.11 | 0.0338 | 0.15 | 1.32 |
| Taurolithocholic acid -3-O-sulfate | 23.82 | 4.39 | 0.0077 | 0.19 | 2.16 |
| Abscisic Acid | 10.45 | 3.14 | 0.0323 | 0.15 | 4.29 |

**Figure S1.** Spearman correlation between polar metabolites, lipid classes, age, gender, BMI, and pharmacological treatment, *i.e.*, ASA/Clopidogrel, Warfarin/NOAC, ACEi/ARB, Calcium flow inhibitor and statins at baseline. *p<0.05
